# Pyrrolidine dithiocarbamate administered during ex-vivo lung perfusion promotes rehabilitation of injured donor rat lungs obtained after prolonged warm ischemia

**DOI:** 10.1371/journal.pone.0173916

**Published:** 2017-03-21

**Authors:** Cyril Francioli, Xingyu Wang, Roumen Parapanov, Etienne Abdelnour, Jérôme Lugrin, Fabrizio Gronchi, Jean Perentes, Philippe Eckert, Hans-Beat Ris, Lise Piquilloud, Thorsten Krueger, Lucas Liaudet

**Affiliations:** 1 Service of Thoracic Surgery, University Hospital Medical Center and Faculty of Biology and Medicine, Lausanne, Switzerland; 2 Service of Adult Intensive Care Medicine, University Hospital Medical Center and Faculty of Biology and Medicine, Lausanne, Switzerland; 3 Service of Anesthesiology, University Hospital Medical Center and Faculty of Biology and Medicine, Lausanne, Switzerland; Emory University School of Medicine, UNITED STATES

## Abstract

Damaged lung grafts obtained after circulatory death (DCD lungs) and warm ischemia may be at high risk of reperfusion injury after transplantation. Such lungs could be pharmacologically reconditioned using ex-vivo lung perfusion (EVLP). Since acute inflammation related to the activation of nuclear factor kappaB (NF-κB) is instrumental in lung reperfusion injury, we hypothesized that DCD lungs might be treated during EVLP by pyrrolidine dithiocarbamate (PDTC), an inhibitor of NF-κB. Rat lungs exposed to 1h warm ischemia and 2 h cold ischemia were subjected to EVLP during 4h, in absence (CTRL group, N = 6) or in presence of PDTC (2.5g/L, PDTC group, N = 6). Static pulmonary compliance (SPC), peak airway pressure (PAWP), pulmonary vascular resistance (PVR), and oxygenation capacity were determined during EVLP. After EVLP, we measured the weight gain of the heart-lung block (edema), and the concentration of LDH (cell damage), proteins (permeability edema) and of the cytokines IL-6, TNF-α and CINC-1 in bronchoalveolar lavage (BAL), and we evaluated NF-κB activation by the degree of phosphorylation and degradation of its inhibitor IκBα in lung tissue. In CTRL, we found significant NF-κB activation, lung edema, and a massive release of LDH, proteins and cytokines. SPC significantly decreased, PAWP and PVR increased, while oxygenation tended to decrease. Treatment with PDTC during EVLP inhibited NF-κB activation, did not influence LDH release, but markedly reduced lung edema and protein concentration in BAL, suppressed TNFα and IL-6 release, and abrogated the changes in SPC, PAWP and PVR, with unchanged oxygenation. In conclusion, suppression of innate immune activation during EVLP using the NF-κB inhibitor PDTC promotes significant improvement of damaged rat DCD lungs. Future studies will determine if such rehabilitated lungs are suitable for in vivo transplantation.

## Introduction

Although lung transplantation is the only definitive treatment available for end-stage lung diseases, this option remains critically limited by the shortage of available donor lungs [1]. Novel strategies, including organ donation after circulatory death (DCD) [2], and the implementation of ex-vivo lung perfusion (EVLP) [3], have recently emerged to overcome such shortage. EVLP was initially developed to assess the function and the potential for transplantation of DCD lungs [4, 5], and its use has been then extended to other forms of “non standard” donor lungs [3]. EVLP has also been proposed as a platform to deliver drugs *ex vivo* (concept of pharmacological reconditioning) [6], in order to improve the status of the donor lung and to reduce the risk of primary graft dysfunction (PGD), a severe form of lung ischemia and reperfusion injury (LIR) which may develop early after transplantation [7]. In line with this concept, we recently provided evidence, in an experimental model of EVLP of DCD lungs, that such lungs could be significantly reconditioned by the *ex vivo* administration of drugs interfering with oxidative processes associated with LIR [8].

A critical pathogenic mechanism of LIR and PGD relies in the rapid activation of innate immune mechanisms responsible for the establishment of an early non specific inflammatory response in the graft [9]. This response is characterized by the upregulated expression of inflammatory cytokines / chemokines, and the stimulated trafficking of activated leukocytes within the transplanted organ [9]. A crucial step in triggering such response relies in the activation of the transcription factor nuclear factor kappaB (NF-κB), a master regulator of inflammation activated in response to the engagement of immune receptors belonging to the interleukin-1 receptor (IL-1R)/Toll-like receptor (TLR) superfamily [10]. Accordingly, pharmacological inhibitors of NF-κB could have potential therapeutic activity in LIR and PGD after lung transplantation [11]. In an earlier study, Ross et al. reported that the selective NF-κB inhibitor pyrrolidine dithiocarbamate (PDTC) reduced lung edema and improved lung function following lung transplantation in a porcine model [12]. This observation raises the hypothesis that PDTC could be a potential candidate drug for the ex-vivo treatment of damaged, non standard lung grafts. In the present work, we addressed this hypothesis by assessing the effects of PDTC administered during EVLP, in damaged rat lungs obtained after circulatory death and extended warm ischemic time.

## Materials and methods

### Animals

Fifteen male Sprague-Dawley rats weighing 300 to 350g (Charles River Laboratory, L’Arbresle, France) were used in this study. All the animals received humane care in compliance with the 'Principles of Laboratory Animal Care' formulated by the National Society for Medical Research and the 'Guide for the Care and Use of Laboratory Animals' prepared by the Institute of Laboratory Animal Resources and published by the National Institutes of Health (NIH Publication No. 86-23, revised 1996). The experiments were approved by our Institutional Animal Care and Use Committee (Service de la consommation et des affaires vétérinaires de l'Etat de Vaud, Authorization Nr. 2637).

### DCD lung graft model

Experiments were conducted using our previously published protocol [8], with slight modifications. Briefly, the animals were anesthetized using inhaled isoflurane for induction and intraperitoneal pentobarbital sodium for maintenance (50mg·kg^-1^ i.p.), tracheotomized and mechanically ventilated with a tidal volume (V_T_) of 7ml·kg^-1^ and a respiratory rate (RR) of 75·min^-1^ (Harvard 683 rodent ventilator). Following a median sternotomy, heparin (600 U) was injected into the right ventricle, and the animals were sacrificed by exsanguination. Perfusion cannulae (Hugo Sachs, Hugstetten, Germany) were inserted into the pulmonary artery (PA) and the left atrium (LA). The chest was left open for one hour at room temperature (warm ischemic time), while the lungs were kept deflated in situ, in order to further enhance the damaging effect of warm ischemia, as previously exposed in detail [8]. The lungs were then flushed with 15ml of cold low-potassium dextran solution (Perfadex®, Xvivo Perfusion, Göteborg, Sweden) through the PA canula, at a perfusion pressure of 20cm H_2_O. After 1h warm ischemia, the lungs were inflated with a volume of 5ml·kg^-1^, at an FiO_2_ of 0.5, and the heart lung blocks were excised and kept 2 hours in 4°C Perfadex solution (cold ischemia), as described [8].

### Ex-vivo lung perfusion and treatment groups

Our EVLP protocol has been previously presented in detail [8]. Briefly, the heart lung block was weighted and mounted into a rat EVLP system (Harvard IL-2 System, Hugo Sachs, Hugstetten, Germany). The left atrium pressure was set at 4cmH_2_O. The ex-vivo perfusion was initiated (flow-controlled mode) using Steen® solution (Xvivo Perfusion, Göteborg, Sweden, pH 7.4), starting at 2% of theoretical cardiac output at a temperature of 10°C, progressively increased over 40 minutes to 7.5% of theoretical cardiac output and 37°C. Ventilation was started after 30min of EVLP, using room air (FiO_2_ 0.21), a RR of 7^.^min^−1^ and a V_T_ of 3 ml^.^kg^−1^, for 10min, then increased to 6 ml^.^kg^−1^, using a Flexivent FX3 ventilator (SCIREQ Inc., Montréal, Canada). The EVLP was maintained for a total of 4 hours. In summary, our DCD lung and EVLP model included 1 h warm ischemia, 2 h cold ischemia and 4h EVLP (for a total preservation time of 7 h). Such model is clinically relevant, as most DCD protocols include a maximum time of warm ischemia < 60 minutes (ideally < 30 minutes) [13], followed by cold preservation of variable duration, and EVLP for 4 to 6h [3, 14] for a total preservation time of 6 to 18 hours [15].

**Fig 1:**
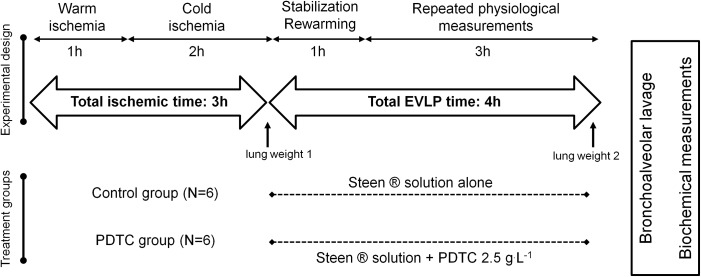
Experimental protocol. Rats were sacrificed by exsanguination and perfusion cannulae were inserted into the pulmonary artery and the left atrium. The chest was left open for one hour at room temperature (warm ischemic time), with the lungs deflated. The lungs were then flushed with cold Perfadex® solution, inflated (5ml·kg^-1^, FiO_2_ 0.5), and kept 2 hours in 4°C Perfadex® (cold ischemic time). The heart-lung blocks were then weighted (lung weight 1) mounted into a rat EVLP system, and perfused for a total of 4 hours, either with Steen® solution alone (Control group, CTRL, N = 6) or with Steen® solution supplemented with pyrrolidine dithiocarbamate ammonium (PDTC, C_5_H_9_NS_2_-NH_3_), at a concentration of 2.5g·L^-1^ (PDTC group, N = 6). At the end of EVLP protocol, the heart-lung blocks were once again weighted (lung weight 2), a bronchoalveolar lavage was performed through the tracheal cannula, and the left lung was frozen for further analysis.

Two experimental groups were evaluated, as graphically depicted in Fig 1. In the first one (control group, CTRL, N = 6), Steen® solution was used as the perfusion solution throughout the study. In the PDTC group, (N = 6), Steen® solution used for the perfusion was supplemented with PDTC ammonium (C_5_H_9_NS_2_-NH_3_; Sigma-Aldrich Chemie GmbH, Buchs, Switzerland), directly dissolved in Steen® solution at a concentration of 2.5g·L^-1^ (15 mmol·L^-1^). This concentration of PDTC was calculated on the basis of previous in vivo administration in experimental rats [16, 17]. Of note, we used a strictly closed perfusion system with constant recirculation (no replenishment with fresh Steen solution during the experiments). At the end of the EVLP protocol, the heart-lung blocks were removed from the system, weighted, and the difference with the initial weight was calculated as an index of lung edema. A bronchoalveolar lavage (BAL) was then performed by instilling 2 ml of phosphate-buffered saline (PBS, pH 7.4) through the tracheal cannula. Recovered BAL fluid was centrifuged (1500g, 10 min, 4°C), and the clear supernatants were then aliquoted and kept at-80°C until further analyses. We did not perform cell counts in the BAL pellets in these experiments. The left lung was then flash frozen and kept at -80°C until further use.

In addition to these two treatment groups, a group of normal rats was sacrificed without any intervention. A BAL was performed and the left lung was then flash frozen. This group of rats served to determine physiological (normal) values for the different biochemical variables measured in our study. Due to the very low variability of these baseline values, this group was limited to N = 3 animals (BASE group).

### Measurements

#### Physiological variables

Pulmonary artery pressure (PAP), left atrial pressure (LAP) and pulmonary flow were continuously recorded, and pulmonary vascular resistance (PVR) was calculated as: PVR = (mean PAP-LAP)/Flow, expressed in cm H_2_O^.^ml^-1.^min^-1^. Peak airway pressure and lung volumes were continuously monitored. After 60 minutes of EVLP (which was the time used to determine baseline values) and every 30 minutes thereafter, static pulmonary compliance was determined during transient and stepwise increases of inspiratory pressure to 15cm H_2_O, according to a programmed function of the FlexVent software unit. Oxygen electrodes (Hugo Sachs Elektronik, Hugstetten, Germany) were placed in the effluent and affluent arms of the EVLP circuit to measure PO_2_.The difference between PO_2_ in both arms was calculated as the oxygenation capacity (ΔpO2) of the lungs.

#### Biochemical variables

**Protein concentration and Lactate Dehydrogenase (LDH) activity in BAL.** Total protein level in BAL was used as a marker of permeability lung edema. It was determined using the Pierce BCA assay (Thermo Scientific, Rockford, USA), and was expressed in mg·ml^-1^ BAL fluid. LDH was measured as an index of cell necrosis, using the Cytotoxicity Detection Kit^PLUS^ from Roche Molecular Biochemicals (Basel, Switzerland), expressed in arbitrary units (AU).

**Concentration of inflammatory cytokines in BAL.** The BAL fluid was assayed to determine the release of the cytokines TNF-α and IL-6, using commercial ELISA kits (Rat TNF-alpha DuoSet, and rat IL-6 Duoset, R&D Systems, Minneapolis, MN, USA) according to the manufacturer’s instructions. BAL was also assayed for the chemokine CINC-1 (Cytokine-Induced Neutrophil Chemoattractant Factor 1, also termed GRO alpha or KC, which is the rat homologue to human interleukin-8), a CXC chemokine (CXCL1) specifically attracting polymorphonuclear cells at sites of inflammation, using the Duoset Rat CXCL1/CINC-1 ELISA kit from R&D System. TNF-α, IL-6, and CINC-1 are expressed in nanogram·ml^-1^ BAL fluid.

**Concentration of Protein Carbonyl adducts in lung tissue.** The formation of protein carbonyl adducts, a marker of oxidative stress [18], was used as an index of oxidative modifications of lung proteins. Frozen lung samples were pulverized in liquid nitrogen, and protein carbonyls were then measured in the tissue powder using an ELISA-based assay according to manufacturer’s instructions (OxiSelect Protein Carbonyl ELISA Kit; Cell Biolabs Inc., San Diego, USA), and expressed in nanomol·mg tissue protein^-1^.

**SDS-PAGE and western immunoblotting.** Tissue powder obtained from frozen lung samples was homogeneized in a RIPA buffer (0.1% SDS, 1% NP-40, 1% sodium deoxycholate, 150 mM NaCl, 50 mM Tris pH 7.4, 1 mM EDTA). Protein concentrations were measured by the BCA assay (Thermo Scientific Pierce, Rockford, IL). Lung proteins (30 μg) were separated by standard SDS-PAGE, and transferred on nitrocellulose membranes. Western immunoblotting was performed using anti-IκBα (Santa Cruz Biotechnology) and anti-phospho-IκBα primary antibodies (Cell Signaling, Beverly, MA) followed by incubation with a HRP-coupled goat-anti rabbit secondary antibody (Jackson ImmunoResearch). Immunoblots were revealed using ECL Western Blotting Reagents (GE Healthcare) and Super RX films (Fujifilm). Signals were quantified using ImageJ software v1.48.

### Statistical analysis

All the results in the study are presented as means ± s.e.m. of *n* observations. Physiological measurements during EVLP were analyzed using 2-way ANOVA for repeated measurements, followed by pairwise comparisons to address the effects of treatment and time (with time T = 60 minutes used as the control) using Bonferroni multiple comparison test. The lung weight gain was compared between the two treatment groups using unpaired *t-*test. For the biochemical measurements, comparisons were made using one-way ANOVA and Tukey’s post-hoc correction. In all comparisons, a p value <0.05 was considered significant. Statistical analyses were performed using Graphpad prism 6 software (GraphPad Software Inc., La Jolla, CA, USA).

## Results

### Biochemical measurements

**Fig 2:**
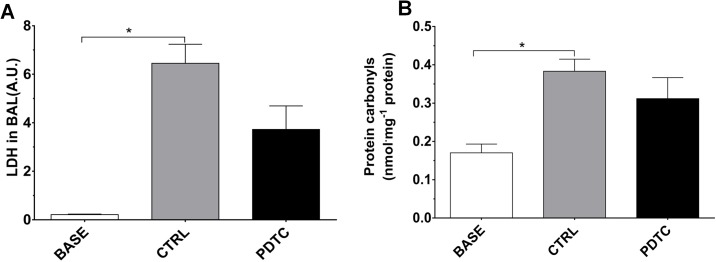
LDH activity in BAL fluid and protein carbonyls in lung tissue homogenates. A. LDH activity in BAL fluid. LDH activity was measured as an index of cellular necrosis, expressed in arbitrary units (A.U.). B: protein carbonyls were measured as an index of oxidative modifications in lung tissue, normalized to the concentration of lung proteins. BASE: BAL fluid and lung tissue were obtained from normal rats sacrificed without any intervention (Normal, baseline values, N = 3); CTRL: Ischemia followed by EVLP with Steen® solution alone (N = 6); PDTC: Ischemia followed by EVLP with PDTC treatment (N = 6). Means ± s.e.m.* p<0.05 (ANOVA followed by Tukey’s test).

LDH and protein carbonyls. The LDH activity in BAL (Fig 2A) was measured as an indicator of cell necrosis. In comparison to normal values (BASE group, 0.21 ± 0.03 AU), a marked increase was noted in the CTRL group (6.45 ± 0.79 AU, p<0.05 vs BASE). In the PDTC group, the LDH activity (3.72 ± 0.97 AU) tended, albeit not significantly, to differ from baseline values (p = 0.06) and from values in the CTRL group (p = 0.08). Protein Carbonyl levels in lung tissue (Fig 2B) were measured as an index of oxidative stress [18]. When compared to the values measured in the normal lungs (BASE group, 0.17 ± 0.02 nmol·ml^-1^), a significant increase of protein carbonyls was noted in the CTRL group (0.38 ± 0.03 nmol·ml^-1^; p<0.05), but not in the PDTC group (0.31 ± 0.05 nmol·ml^-1^; p = 0.12 vs BASE). The difference between CTRL and PDTC did not reach statistical significance.

#### Inflammatory cytokines

**Fig 3:**
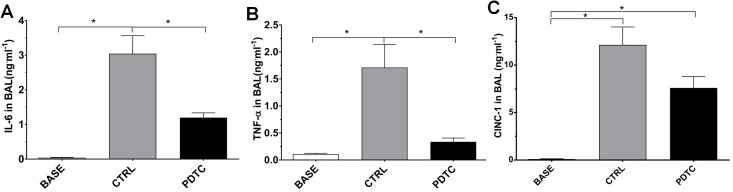
Concentrations of inflammatory cytokines in the BAL fluid. BAL fluid concentration of IL-6 (A), TNFα (B) and CINC-1 (C), expressed in ng^.^ml^-1^ BAL fluid. BASE group: BAL fluid was obtained from normal rats sacrificed without any intervention (normal values, N = 3); CTRL: Ischemia followed by EVLP with Steen® solution alone (N = 6); PDTC: Ischemia followed by EVLP with PDTC treatment (N = 6). Means ± s.e.m.* p<0.05. (ANOVA followed by Tukey’s test).

The concentrations of cytokines measured in the BAL are depicted in Fig 3. With respect to IL-6 (Fig 3A), its basal concentration, as measured in the BASE group, was very low (0.05 ± 0.02 ng·ml^-1^). After EVLP, IL-6 was markedly elevated in the CTRL group (2.97 ± 0.48 ng·ml^-1^), but was significantly reduced by PDTC (1.15 ± 0.13 ng·ml^-1^, p<0.05). A similar pattern was noted for TNFα (Fig 3B), whose levels were very low in the normal lungs (BASE group, 0.11 ± 0.02 ng·ml^-1^). Following EVLP, TNFα was markedly increased in the CTRL group (1.71 ± 0.43 ng·ml^-1^), and this increase was significantly prevented in the PDTC group (0.34 ± 0.07 ng·ml^-1^, p<0.05). Finally, the levels of CINC-1 (Fig 3C), almost undetectable in the BASE group (0.14 ± 0.06 ng·ml^-1^), were elevated after EVLP in both the CTRL (11.38 ± 1.25 ng·ml^-1^) and PDTC groups (7.4 ± 1.23 ng·ml^-1^). Although this increase was less pronounced in the PDTC group, the difference with the CTRL group did not reach statistical significance.

#### Heart-lung weight gain and protein concentration in BAL fluid

**Fig 4:**
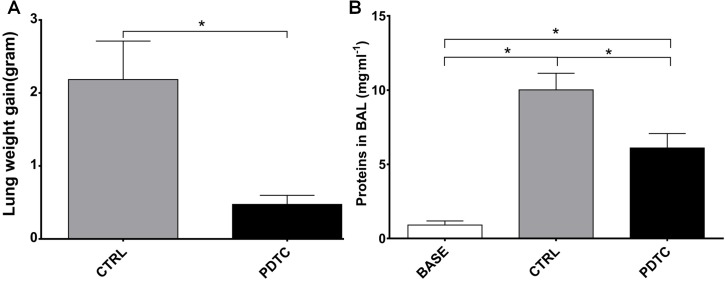
Indices of lung edema. A. Weight gain of the heart-lung blocks expressed in grams, represents the difference between weight of the heart-lung blocks measured before and at the end of EVLP. B. The concentration of proteins in the BAL fluid (in mg^.^ml^-1^ BAL fluid) was measured as an index of high permeability edema. BASE: BAL fluid and was obtained from normal rats sacrificed without any intervention (Normal, baseline values, N = 3); CTRL: Ischemia followed by EVLP with Steen® solution alone (N = 6); PDTC: Ischemia followed by EVLP with PDTC treatment (N = 6). Means ± s.e.m.* p<0.05 (Heart-lung block weight gain: unpaired *t* test; Proteins in BAL fluid: ANOVA followed by Tukey’s test).

We measured the change in weight of the heart-lung blocks before and at the end of EVLP as an index of edema accumulation. As indicated in Fig 4A, the weight change (weight gain) reached 2.19 ± 0.53 g in the CTRL group, whereas it was only of 0.47 ± 0.05 g in the PDTC group, a statistically significant difference (p<0.05). We also measured the concentration of proteins in the BAL fluid as an index of high permeability pulmonary edema (Fig 4B). In comparison to very low normal values measured in rats sacrificed without any further intervention, both groups subjected to EVLP disclosed significant increased protein content in the BAL fluid, but the increase was significantly more pronounced in the CTRL group (10.02 ± 1.12 mg·ml^-1^) than in the PDTC group (6.1 ± 0.97mg·ml^-1^; p<0.05 vs CTRL).

### Physiological variables

#### Static pulmonary compliance and peak airway pressure

**Fig 5:**
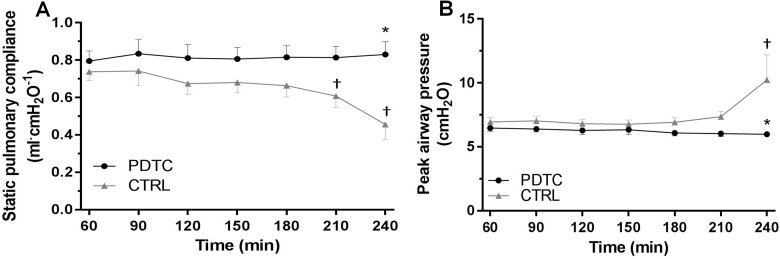
Static pulmonary compliance and peak airway pressure during EVLP. Static pulmonary compliance (A) and peak airway pressure (B) were computed 60 minutes after EVLP initiation (baseline values), and every 30 minutes thereafter. CTRL: Ischemia followed by EVLP with Steen® solution alone (N = 6); PDTC: Ischemia followed by EVLP with PDTC treatment (N = 6). Means ± s.e.m. † p < 0.05 vs baseline value; * p<0.05 PDTC vs CTRL (two way ANOVA with Bonferroni’s adjustments for multiples comparisons)

**Fig 6:**
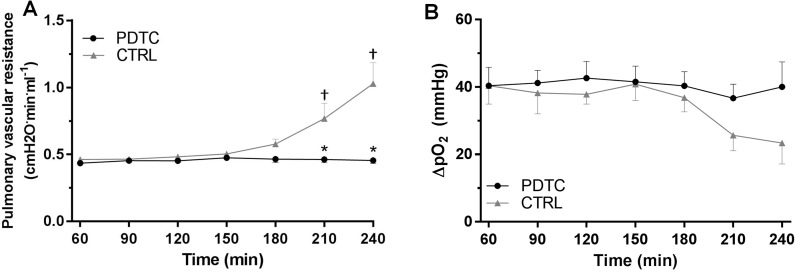
Pulmonary vascular resistance and oxygenation index during EVLP. Pulmonary vascular resistance (A) was calculated as (mean pulmonary artery pressure - left atrial pressure)/perfusion flow). The oxygenation capacity (B) was calculated as the difference in the partial pressure of O_2_ between the effluent and the affluent arms of the EVLP circuits. The vascular resistance and oxygenation capacity were computed after 60 minutes of EVLP (baseline values), and every 30 minutes thereafter. Means ± s.e.m. † p < 0.05 vs baseline value; * p<0.05 PDTC vs CTRL (two way ANOVA with Bonferroni’s adjustments for multiples comparisons).

The physiological data recorded during the 4 h of EVLP are depicted in Figs 5 and 6. In the CTRL group, the static pulmonary compliance (SPC, Fig 5A) progressively decreased during the study, the difference with baseline values reaching statistical significance at the end of the experimental period (SPC = 0.74 ± 0.05 ml·cmH_2_O^-1^ at 60min; 0.61 ± 0.06 ml·cmH_2_O^-1^ at 210min; 0.46 ± 0.08ml·cmH_2_O^-1^ at 240min, p<0.05 vs 60 min). By contrast, SPC remained stable in the PDTC group (SPC = 0.80 ± 0.06 ml·cmH_2_O^-1^ at 60min; 0.83 ± 0.07 ml·cmH_2_O^-1^ at 240min), and was therefore statistically higher than in the CTRL group at the end of the study (240 min). Peak airway pressure (PAWP, Fig 5B) displayed a significant increase only in the CTRL group at time 240 min (PAWP = 6.94 ± 0.38 cmH_2_O at 60min; 10.23 ± 1.97 cmH_2_O at 240min, p<0.05), whereas it remained stable in the PDTC group (PAWP = 6.47 ± 0.26 cmH_2_O at 60min; 5.98 ± 0.22 cmH_2_O at 240min), the difference between the two groups being significant at 240min (p<0.05).

#### Pulmonary vascular resistance and oxygenation capacity

Pulmonary vascular resistance (PVR, Fig 6A) significantly increased at 210 and 240 minutes in the CTRL group (PVR = 0.46 ± 0.02 cmH_2_O·min·ml^-1^ at 60min; 0.77 ± 0.12 cmH_2_O·min·ml^-1^ at 210min; 1.03 ± 0.16 cmH2O·min·ml^-1^ at 240min, p<0.05 vs 60 min), but not in the PDTC group (PVR = 0.44 ± 0.02 cmH2O·min·ml^-1^ at 60min and 0.46 ± 0.02 cmH2O·min·ml^-1^ at 240min, p<0.05 vs CTRL). With respect to the oxygenation capacity (ΔpO_2_, Fig 6B), there was a trend towards progressive deterioration in the CTRL group (40.4 ± 5.5 mmHg at 60min; 23.4 ± 6.2mmHg at 240min, p = NS vs 60 min), but no changes in the PDTC group (40.4 ± 5.5 mmHg at 60min; 40 ± 7.4 mmHg at 240min), but the differences did not reach statistical significance.

### NF-κB activation in lung tissue

**Fig 7:**
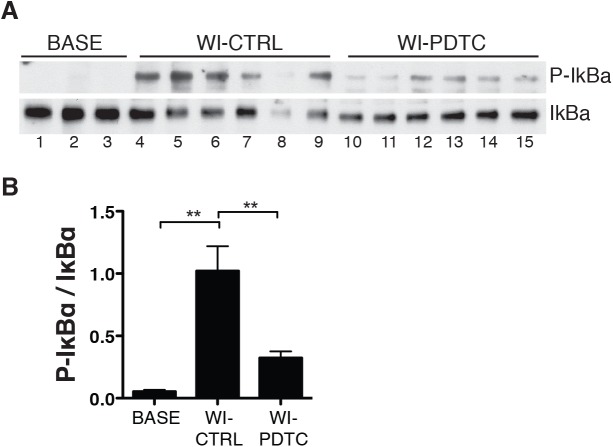
Phosphorylation and degradation of IκBα in lung tissue. Western immunoblots of IκBα and phospho-IκBα in all lungs (A). The graph depicts the ratio of the densitometric analysis of phospho-IκBα and IκBα (B). Means ± s.e.m; ** p<0.01 (ANOVA followed by Tukey’s test).

The critical step in NF-κB activation relies in the phosphorylation of its cytoplasmic inhibitor IκBα, followed by its polyubiquitination and dedradation in the proteasome. As indicated in Fig 7, both IκBα phosphorylation and IκBα degradation (Fig 7A) were found in the control (untreated) group, with an elevated ratio of phospho IκBα/ IκBα on densitometric analysis (Fig 7B), indicative of NF-κB activation. In contrast, lungs treated with PDTC had more preserved levels of IκBα, so that the ratio phospho IκBα/ IκBα was significantly reduced, in agreement with the known mechanism of NF-κB inhibition by PDTC, which relies in the prevention of IκBα degradation [19].

## Discussion

The main results of this study are that the NF-κB inhibitor pyrrolidine dithiocarbamate (PDTC), administered during EVLP of rat lungs obtained after circulatory death and warm ischemia, significantly reduced lung inflammation, edema formation and physiological deterioration. These findings support that pharmacological interference with the innate immune response may represent a useful option for the treatment of damaged lung grafts.

Many potentially injured donor lungs are rejected for transplantation, due mainly to their marked sensitivity to reperfusion injury, which may result in primary graft dysfunction (PGD) after transplantation with significant negative impact on outcomes [20]. In this context, assessment and ex vivo treatment of such damaged lungs using EVLP clearly represents a breakthrough to increase the pool of suitable donor lungs [15]. EVLP may notably be of major interest for the evaluation of lungs obtained after cardiac arrest (DCD lungs), which are at higher risk of injury and deterioration [6]. Interestingly, EVLP on its own appears to have a positive influence on the outcome of transplanted DCD lungs, as indicated by shorter hospital stay and trends towards reduced PGD in human recipients of DCD lungs with EVLP vs no EVLP [15]. This effect may be related to the optimal oncotic pressure of the perfusion solution, which might reduce the development of pulmonary edema during reperfusion [6].

This notwithstanding, it is also important to indicate that significant reperfusion/reoxygenation injury of the donor lung can occur during EVLP [8], which may result, in severe forms, in the progressive deterioration of the graft, which must finally be discarded for transplantation [3, 15]. DCD lungs are at higher risk for such deterioration, due to the unavoidable warm ischemic period occurring between cardiac arrest and organ procurement [8, 21]. In such conditions, EVLP may offer the unique opportunity to deliver drugs able to interfere with the process of reperfusion injury and thus improve the status of the potential donor graft [22, 23]

Innate immune activation is key to the development of reperfusion injury [24], and as such, is instrumental in the setting of lung transplantation [9]. During reperfusion, free radicals and oxidants are formed as a result of reoxygenation, leading to oxidative tissue injury [25]. Damaged cells and extracellular matrix then release endogenous molecules, termed damage-associated molecular patterns (DAMPs), which foster a sterile inflammatory response after binding to innate immune receptors, primarily the toll-like receptors (TLRs) [26]. TLRs, especially TLR2 and TLR4, elicit intracellular signaling through the adaptor molecule MyD88, to activate the transcription factor NF-κB [27], which can be further activated by oxidants and free radicals, owing to its redox-sensitive nature [28]. NF-κB in turn orchestrates the inflammatory response, by triggering the expression of a wealth of pro-inflammatory cytokines and other mediators [27]. NF-κB activation was well demonstrated in the present study, as indicated by the phosphorylation and degradation of the NF-κB inhibitor IκBα. Such degradation represents the critical step in the process of NF-κB activation, as it allows NF-κB subunits to translocate from the cytoplasm into the nucleus [28]. It is likely that NF-κB activation occurred not only in prototypical immune cells, primarily resident macrophages, but also in lung parenchymal cells, which disclose strong innate immune activation during lung inflammatory processes [29]. In agreement with its known pharmacological actions [19], PDTC significantly inhibited these processes, confirming its potent activity as an inhibitor of NF-κB signaling.

The importance of NF-κB activation in the pathophysiology of ischemia-reperfusion supports the notion that manipulating NF-κB-dependent signaling might reduce inflammatory damage in this setting[30]. With specific respect to the lung, Altemeier et al. recently reported that mice genetically deficient in MyD88, the main adaptor in the NF-κB cascade, had reduced lung edema and inflammatory cytokine expression after in vivo unilateral lung ischemia-reperfusion [31]. In a study of lung transplantation in pigs, Ross and co-workers found reduced lung edema and improved oxygenation of the transplanted lung, when NF-κB was inhibited using PDTC, added to the preservation solution at the time of organ procurement [12]. Whether such approach would allow ex-vivo treatment of an injured donor lung has however never been studied, an issue that was the central hypothesis of the present work, addressed in a clinically-relevant model of EVLP [8].

Lungs retrieved after 1h warm ischemia followed by 2h cold ischemia and 4h EVLP displayed significant damage, evidenced by massive release of LDH, edema formation with marked protein permeability and severe functional impairment. We recently reported that oxidative stress is a central mechanism of lung injury in this setting, as outlined by the considerable protection afforded by the potent antioxidant MnTBAP during EVLP [8]. In the present study, PDTC did not significantly reduced oxidative stress, indicated by the only modest reduction of protein carbonyls. As a result, the release of LDH, a strong indicator of oxidant-mediated cellular damage [32], was only partially, but not significantly, reduced by PDTC. Although PDTC possesses some antioxidant properties related to its ability to chelate iron and inhibit the Fenton reaction [33], our findings indicate, therefore, that such an effect did not prevail in our experimental conditions.

The central observation of the present study was the prominent inflammatory response which occurred in untreated lungs and which was largely mitigated by PDTC. This response was characterized by the release of large amounts of the cytokines IL-6, TNFα, and the chemokine CINC-1 (rat homologue of human IL-8), into the BAL fluid. It is particularly noticeable that these same cytokines have been shown to play major pathogenic roles in human lung transplantation, with elevated levels measured in pleural fluid, plasma or BAL fluid [34–36] after transplantation, higher levels being correlated with worse outcomes [36, 37]. Furthermore, it is worth mentioning that a recent study pointed out significant increases of IL-6 and IL-8 in human lungs undergoing EVLP [38]. Cellular sources of these cytokines in the lung are probably multiple, including not only resident immune cells, but also parenchymal cells, primarily alveolar epithelial cells, which can notably produce large amounts of chemokines such as CINC-1/IL-8 [29, 39]. Our finding that PDTC attenuated the release of cytokines into the BAL fluid is in total agreement with its inhibitory actions on NF-κB and immune activation [33]. Importantly, we administered PDTC after the period of ischemic storage, implying that innate immune activation occurred during the process of reperfusion and reoxygenation, which takes place during EVLP. This observation has obvious clinical relevance, for it emphasizes that EVLP offers a time window for therapeutic interventions well after lung procurement, but before transplantation, which further validates the concept of pharmacological reconditioning of damaged donor lungs using EVLP.

The dampened inflammatory response afforded by PDTC was associated with a considerable reduction of lung edema at the end of EVLP, characterized by reduced lung water accumulation and BAL protein concentration. These findings emphasize the crucial role of inflammatory cytokines in promoting epithelial and endothelial disruption leading to high permeability pulmonary edema. TNFα promotes lung barrier dysfunction (reviewed in [40]) via cytotoxic actions towards lung microvascular endothelial cells [41] and by disrupting endothelial permeability through microtubule destabilization [42]. In addition, TNFα impairs the clearance of alveolar liquid by reducing the expression of the sodium channel ENaC in lung epithelial cells [43]. IL-6, which can increase endothelial permeability by altering the ultrastructural distribution of tight junctions [44], has been shown to be a potent mediator of alveolar-capillary barrier disruption in experimental models of lung inflammation [45, 46]. These deleterious effects of IL-6 on pulmonary permeability may occur in the absence of inflammatory cell recruitment [45, 46], which is highly relevant to our experimental conditions, given the acellular perfusate that was used during EVLP.

In line with the development of severe pulmonary edema, lungs from control rats displayed a significant physiological deterioration during the last hour of EVLP. This was evidenced by a rapid increase of airway pressure together with a decline of pulmonary compliance, as well as a steady rise of pulmonary vascular resistance. Remarkably, PDTC-treated lungs disclosed an outstanding stability of airway pressure and lung compliance, which is very likely to be imputed to the reduced lung edema provided by PDTC, owing to the mechanical stress imposed by liquid-filled alveoli on adjacent air-filled alveoli in the presence of pulmonary edema [47]. Also, PDTC treatment prevented the rise in vascular resistance, suggesting reduced endothelial dysfunction with less vasoconstriction in the pulmonary vascular bed during EVLP, consistent with the downregulation of inflammatory cytokines.

Although the oxygenation capacity tended to decline at the end of EVLP in the control group, this effect was not significant, and was only marginally improved by PDTC. This observation may appear surprising, given the significant pulmonary edema that should have resulted in an impaired oxygen transfer. However, as mentioned in our previous work, such lack of influence on PO_2_ may be largely related to the use of an acelluar perfusate in our EVLP protocol [8]. In such medium, the PO_2_/O_2_ content relationship is linear, so that the shunted perfusate leaving poorly aerated alveoli has only a very small and nonsignificant influence on the PO_2_ of the effluent. This is entirely consistent with findings by Yeung et al [48], who reported in a model of porcine EVLP, that true shunt affected effluent PO_2_ only when using a cellular, but not an acellular perfusate [48]. For this reason, it has been suggested that physiological variables such as compliance, airway pressure, and vascular resistance may be more useful than oxygenation to evaluate graft function during EVLP [48, 49].

Our study has some limitations. Firstly, we did not include a control group of normal lungs undergoing EVLP to determine the intrinsic effects of EVLP in the absence of warm ischemia. However, it is here worth to mention that in our recent study [8], we showed that lungs not exposed to warm ischemia (but simply to cold preservation) disclosed no significant lung damage and edema, while showing remarkable physiological stability throughout EVLP, implying that EVLP itself does not produce significant alterations. Secondly, lungs treated during EVLP were not transplanted. Therefore, the significant improvement of injured lungs by PDTC noted during EVLP cannot be translated to transplantated lungs, and this issue will clearly require further in vivo transplantation studies. Thirdly, we did not perform histological analyses to evaluate the actions of PDTC on lung morphology, nor did we perform any cell counts and cellular analyses in the BAL fluid. However, it must be underscored that the key morphological consequence of lung reperfusion injury and inflammation is the infiltration of alveolar spaces by blood leukocytes. Owing to the acellular perfusate used in our study, such infiltration cannot be evaluated [8], although we cannot rule out the presence of resident lung cells, including some leukocytes. This issue will also require further in vivo studies. Finally, as we computed the weight gain of the heart-lung blocks as an index of lung edema, we cannot formally rule out some contribution of changes in heart weight in our observations. In this respect, comparing the lung dry/wet weight ratio of the groups at the end of experiments would have been more accurate.

In conclusion, our study indicates that the NF-κB inhibitor pyrrolidine dithiocarbamate, administered during ex-vivo perfusion of injured rat lungs obtained after circulatory death, markedly alleviates pulmonary inflammation and edema, thereby preventing physiological deterioration. Therefore, pharmacological therapy with PDTC during EVLP may be useful for the rehabilitation of damaged donor lungs before transplantation.
